# Endoscopic Diagnosis of Leiomyosarcoma of the Esophagus, a Rare Neoplasm

**DOI:** 10.1155/DTE.5.49

**Published:** 1998

**Authors:** Mario Ravini, Massimo Torre, Giulio Zanasi, Marco Vanini, Mario Camozzi

**Affiliations:** 1 Divisione di Chirurgia Toracica Azienda Ospedaliera Niguarda CàGranda P.za Ospedale Maggiore 3/5 Milano 20162 Italy; 2 Service of Gastroenterology and Digestive Endoscopy Italy; 3 Insitute of Patholbgy Ospedale Niguarda CàGranda Milano Italy

## Abstract

We report a case of leiomyosarcoma of the distal third of the esophagus in a 51-year-old woman presenting with a six-month history of severe epigastric pain, disphagia and weight loss. The diagnosis, suspected on endoscopic examination, was preoperatively acheived by biopsy and immunohistological stain. Surgical treatment was undertaken with good results. Differentiation between leiomyosarcoma and more common esophageal neoplasm may be difficult if based on radiographic and endoscopic appearance. Preoperative histological confirmation is therefore mandatory to schedule a wide surgical excision.


*Diagnostic and Therapeutic Endoscopy*, Vol. 5, pp. 49–52
Reprints available directly from the publisher
Photocopying permitted by license only

© 1998 OPA (Overseas Publishers Association) N.V.
Published by license under
the Harwood Academic Publishers imprint,
part of The Gordon and Breach Publishing Group.
Printed in Singapore.

### Endoscopic Diagnosis of Leiomyosarcoma of the Esophagus, a Rare Neoplasm

MARIO RAVINI[a,*], MASSIMO TORRE[a], GIULIO ZANASI[b], MARCO VANINI[c] and MARIO CAMOZZI[c]

[a]*Divisione di Chirurgia Toracica, Azienda Ospedaliera Niguarda CàGranda, P.za Ospedale Maggiore 3/5 20162 Milano, Italy;* [b]*Service of Gastroenterology and Digestive Endoscopy,* [c]*Insitute of Pathology, Ospedale Niguarda, CàGranda, Milano, Italy*

*(Received 1 February 1998; In final form 16 March 1998)*

**We report a case of leiomyosarcoma of the distal third of the esophagus in a 51-year-old woman presenting with a six-month history of severe epigastric pain, disphagia and weight loss. The diagnosis, suspected on endoscopic examination, was preoperatively acheived by biopsy and immunohistological stain. Surgical treatment was undertaken with good results. Differentiation between leiomyosarcoma and more common esophageal neoplasm may be difficult if based on radiographic and endoscopic appearance. Preoperative histological confirmation is therefore mandatory to schedule a wide surgical excision.**

*Keywords:* Leiomyosarcoma, Esophageal neoplasms, Esophagus, Endoscopy, Surgery

### INTRODUCTION

Leiomyosarcoma of the esophagus is a very unusual neoplasm accounting for 0.1–0.5% of all esophageal malignancies [1–3]. Preoperative diagnosis is difficult in the majority of the cases owing to its radiological and endoscopic appearance mimicking other more frequent esophageal lesions. Recently we have successfully treated a patient in whom an esophageal leiomyosarcoma was preoperatively suspected on the basis of endoscopic appearance and histologically confirmed by immunoreactive stain.

### CASE REPORT

A 51-year-old heavy smoker woman, with a six-month history of epigastric pain, disphagia and weight loss, was admitted at our institution on April 10, 1997. Past history showed tonsillectomy at the age of 12 years and appendectomy at the age of 16 years. Physical examination revealed epigastric pain exacerbated by manual palpation. Routine laboratory tests, serum level of CEA and TPA were within normal limits. Barium swallow showed a filling defect in the middle third of the viscus.

* Corresponding author. Tel./Fax: +39 02 6444 2271.

Esophagoscopy confirmed an intraluminal lobulated mass without ulceration, extending 29–35 cm from the incisors. The mass was pedunculated, with a wide implantation site on the anterior wall of the esophagus and it obliterated the lumen almost completely.

Several specimens were obtained during endoscopy. The formalin fixed and paraffin embedded samples were cut at 5 μm thickness. The slices, stained with H-E, showed a biphasic population composed partly by cells with elongated eosinophilic cytoplasm, with blunt-end, "cigar shaped" nuclei arranged in cartwheel pattern and partly by multinucleated and/or giant cells with highly pleomorphic nuclei (Fig. 1). Most cells showed a clear-cut immunoreactivity for smooth muscle alpha actin and more than 60% of the cells showed positivity for MIB-1 (Fig. 2). Morphology and immunoreactive patterns were highly consistent with the diagnosis of primitive leiomyosarcoma (Fig. 3).

Computed tomography of the chest and upper abdomen excluded distant metastasis and mediastinal node enlargement. Fiberoptic bronchoscopy excluded tracheal compression and/or infiltration. On April 16, 1997 the patient underwent Ivor–Lewis esophagocardiectomy with esophagogastrostomy above the azygos vein via a right posterolateral thoracotomy and an upper midline laparotomy. Postoperative course was uneventful and the patient was discharged on the 9th postoperative day. At nine months after the operation the patient is symptom free, has gained 5 kg and has no evidence of recurrence.

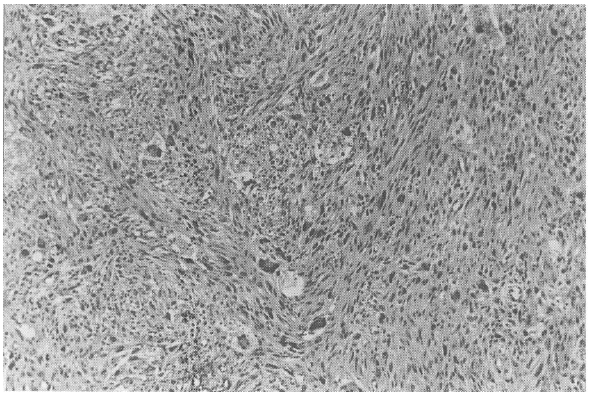

FIGURE 1 Esophageal leiomyosarcoma: microscopic aspect of endoscopic biopsy specimen. Haematoxilyn & Eosin 100×.

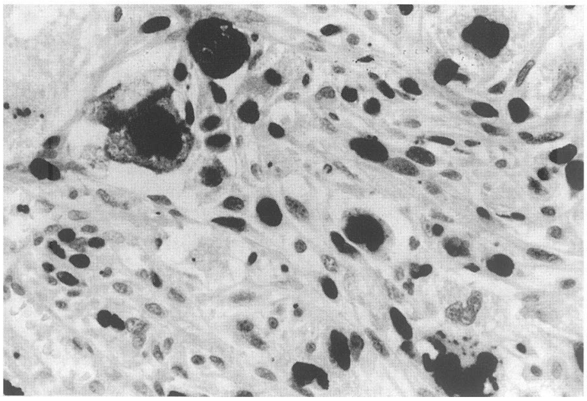

FIGURE 2 Esophageal leiomyosarcoma: strong $\alpha$-actin immunoreactivity demonstrating the smooth muscle differentiation of the neoplasm (400×).

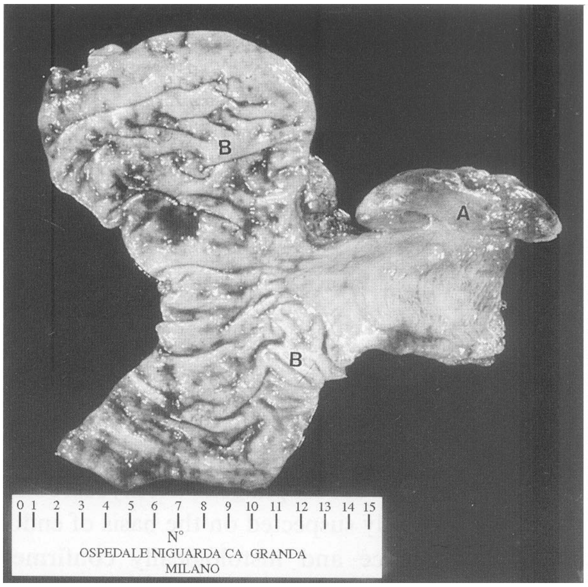

FIGURE 3 Esophageal leiomyosarcoma: gross appearance of the pedicled mass protruting from esophageal mucosa (A) with haemorrhagic areas; (B) gastric mucosa.

### PATHOLOGICAL FINDINGS

On gross inspection the tumor consisted of a mass measuring 8 × 5 × 3 cm joined to the underlying esophageal mucosa by a short pedicle. Both the outer and the cut surfaces were fleshy, white-grey with small superficial tan areas due to haemorrhage (Fig. 3). Microscopic examination confirmed the preoperative diagnosis. No infiltration of the underlying esophageal mucosa was demonstrated. The resection margins were tumor free and the adjacent lymph nodes were normal.

### DISCUSSION

In 1902 Howard described a case of "primary sarcomas of the esophagus" [4]. Since then nearly 100 cases have been reported in English literature so far. The present case did not substantially differ from those previously described as for age and clinical features. The tumor was located in the distal third of the esophagus and presented as a fusiform polypoid mass, which was responsibe for dysphagia and epigastric pain. The patient's prolonged smoke abuse (3 packs/year for 30 years) without a history of exposure to other known carcinogens is noteworthy. No mention is found in the literature about the relationship between carcinogens and esophageal leiomyosarcoma. Barium meal study and esophagoscopy are essential but not specific for the diagnosis. When the tumor shows a submucosal growth, it may be misdiagnosed as leiomyoma and when it presents as polypoid mass with ulcerations or erosions as in the case described by Koga *et al.* [5], it may simulate a squamous cell carcinoma. For this reason the diagnostic yield of endoscopic biopsy depends on the gross appearance of the tumor. Granmayeh *et al.* [6] and Ramer *et al.* [7] described the characteristic angiographic findings of a leiomyosarcoma, which do not substantially differ from those of leiomyomas. Aimoto *et al.* [8] first described the typical endoscopic ultrasonic findings (EUS) of esophageal leiomyosarcoma, but further study is needed to define the role of EUS in the diagnosis of the tumor. In our patient the diagnosis of leiomyosarcoma was suspected on the basis of endoscopic appearance and confirmed histologically owing to the immunoreactivity for smooth muscle alpha actin and the elevated positivity for MIB-1. Moreover, in agreement with Pesarini *et al.* [9] histopathological grading of the tumor as low-grade or high-grade sarcomas in dependance of the number of the mitosis may have some prognostic value.

The treatment of choice of leiomyosarcoma of the esophagus is surgical. Survival curve, in the series reviewed by Koga *et al.* [5], shows that 1-, 3-, and 5-year survival rates are 60.3%, 42.8% and 32.1%, respectively. Although therapies, location and size of the tumor do not influence the survival rate, it has been reported that female gender and polypoid growth are better prognostic factors [10]. Visioli *et al.* [11] reported a case who survived 22 years from the original treatment. In the experience of Shiraishi *et al.* [12] metastatic bone localization was found 14 months after operation in a 73 year-old man. Chemotherapy was ineffective and the patient died 3 years after operation.

In conclusion when at esophagoscopy a polypoid mass is found, it must be taken into account the possibility of a leiomyosarcoma. Every effort must be done to achieve a correct preoperative diagnosis, by means of biopsies, particularly when the mucosa is not ulcerated, in order to avoid endoscopic resection and to schedule a wide surgical excision of the tumor.

### *References*

[1] Almeida, J.M.M. Leiomyosarcoma of the esophagus. *Chest* 1982; **81**: 761–763.
[2] Athanasoulis, C.A. and Aral, I.M. Leiomyosarcoma of the esophagus. *Gastroenterology* 1968; **54**: 271–274.
[3] Choh, J.H., Khazei, A.H. and Ihm, H.J. Leiomyosarcoma of the esophagus: report of a case and review of the literature. *J. Surg. Oncol.* 1986; **32**: 223–226.
[4] Howard, W.T. Primary sarcoma of the esophagus and stomach. *JAMA* 1902; **38**: 392–399.
[5] Koga, H., Iida, M., Suekane, H. *et al.* Rapidly growing esophageal leiomyosarcoma: case report and review of the literature. *Abdom. Imaging* 1995; **20**: 15–19.

[6] Granmayeh, M., Jonsson, K., McFarland, W. *et al.* Angiography of abdominal leiomyosarcoma. *AJR* 1978; **130**: 725–730.

[7] Ramer, M., Mitty, M.A. and Baron, M.G. Angiography in leiomyomatous neoplasms of the small bowel. *AJR* 1971; **113**: 263–268.

[8] Aimoto, T., Sasajima, K., Kyono, S. *et al.* Leiomyosarcoma of the esophagus: report of a case and preoperative evaluation by CT scan, endoscopic ultrasonography and angiography. *Gastroenterol. Jpn.* 1992; **27**: 773–779.

[9] Pesarini, A.C., Ernst, H., Ell, C. *et al.* Leiomyosarcoma of the esophagus: Case report, clinicopathological feature, diagnosis and management. *Med. Klin.* 1997; **92**: 234–241.

[10] Choh, J.H., Khazei, A.H. and Ihm, H.J. Leiomyosarcoma of the esophagus: report of a case and review of the literature. *J. Surg. Oncol.* 1986; **32**: 223–226.

[11] Visioli, A. and Daniel, F.J. Leiomyosarcoma of the oesophagus: a case report and literature review of leiomyosarcoma. *Australasian Radiology* 1997; **41**(2): 160–165.

[12] Shiraishi, M., Takahashi, T., Yamashiro, M. *et al.* A report of leiomyosarcoma of the esophagus. *Jpn. J. Geriatrics.* 1995; **32**: 286–291.

